# Integrating resource defence theory with a neural nonapeptide pathway to explain territory-based mating systems

**DOI:** 10.1186/1742-9994-12-S1-S16

**Published:** 2015-08-24

**Authors:** Ronald G Oldfield, Rayna M Harris, Hans A Hofmann

**Affiliations:** 1 Texas Research Institute for Environmental Studies, Sam Houston State University, Huntsville, TX 77341 USA; Department of Biology, Case Western Reserve University, Cleveland, OH 44106 USA; 2 Department of Integrative Biology, The University of Texas at Austin, Austin, TX 78712 USA; 3 Institute for Cellular and Molecular Biology, The University of Texas at Austin, Austin, TX 78712 USA; 4 Institute for Neuroscience, The University of Texas at Austin, Austin, TX 78712 USA

**Keywords:** AVP, AVT, monogamy, polygyny, territory, V1a

## Abstract

The ultimate-level factors that drive the evolution of mating systems have been well studied, but an evolutionarily conserved neural mechanism involved in shaping behaviour and social organization across species has remained elusive. Here, we review studies that have investigated the role of neural arginine vasopressin (AVP), vasotocin (AVT), and their receptor V1a in mediating variation in territorial behaviour. First, we discuss how aggression and territoriality are a function of population density in an inverted-U relationship according to resource defence theory, and how territoriality influences some mating systems. Next, we find that neural AVP, AVT, and V1a expression, especially in one particular neural circuit involving the lateral septum of the forebrain, are associated with territorial behaviour in males of diverse species, most likely due to their role in enhancing social cognition. Then we review studies that examined multiple species and find that neural AVP, AVT, and V1a expression is associated with territory size in mammals and fishes. Because territoriality plays an important role in shaping mating systems in many species, we present the idea that neural AVP, AVT, and V1a expression that is selected to mediate territory size may also influence the evolution of different mating systems. Future research that interprets proximate-level neuro-molecular mechanisms in the context of ultimate-level ecological theory may provide deep insight into the brain-behaviour relationships that underlie the diversity of social organization and mating systems seen across the animal kingdom.

## Introduction

Understanding the causes and consequences of animal behaviour is a fundamental goal in biology [1]. Advances in behavioural ecology, especially during the last three decades of the 20^th^ century, provide an ultimate-level understanding of how ecological and evolutionary forces shape behaviour and social organization (e.g., [2]). At a more proximate level, advances in neuroendocrinology have increased our understanding of the physiological and molecular mechanisms that mediate changes in behaviour in response to ecological and social stimuli [3-6]. However, a conceptual integration of ultimate- and proximate-level perspectives is yet lacking but may provide a more complete understanding of how neuroendocrine mechanisms mediate variation in behaviour and social organisation [7].

In this review we explore the thesis that neural expression of the nonapeptides arginine vasopressin (AVP), vasotocin (AVT) and their receptor V1a may facilitate territoriality, which in turn may shape mating systems in diverse taxa. First, we provide a framework rooted in resource defence theory to illustrate the relationships among aggression, territoriality, and mating system. Next, we consider the relationship between neural AVP, AVT, and V1a and aggression and territoriality and then gather and assess published studies that have examined AVP, AVT, and V1a variation within particular species. We find that neural vasopressinergic and vasotocinergic action is more closely associated with territoriality than it is with outright aggression. Then, we argue that resource defence theory could explain evolution in a neural vasopressinergic or vasotocinergic circuit at the species level, and we review studies that have compared neural AVP, AVT, and V1a between species that differ in territorial behaviour. Because territoriality is a component of the amalgamation of behaviour that we call mating system, we conclude that selection for up-regulation of a neural vasopressinergic or vasotocinergic circuit could mediate territorial behaviour and therefore play a role in the evolution of different resource-based mating systems.

## Aggression, territoriality, mating systems

There are many reasons why animals may behave aggressively, and one of the most common reasons is to defend resources [8] such as food [9], shelter [10], and mates and offspring [11]. In contrast, costs of defence might include injury, vulnerability to predators, time spent not exploiting the resource, and energy expended [12]. The decision to defend a resource depends on benefits - costs, which depends on the resource's distribution in space and time [9], on the individual's motivation and its body condition, and on other ecological factors such as number of competitors, available space, habitat complexity, and predation threat [13-16].

**Figure 1:**
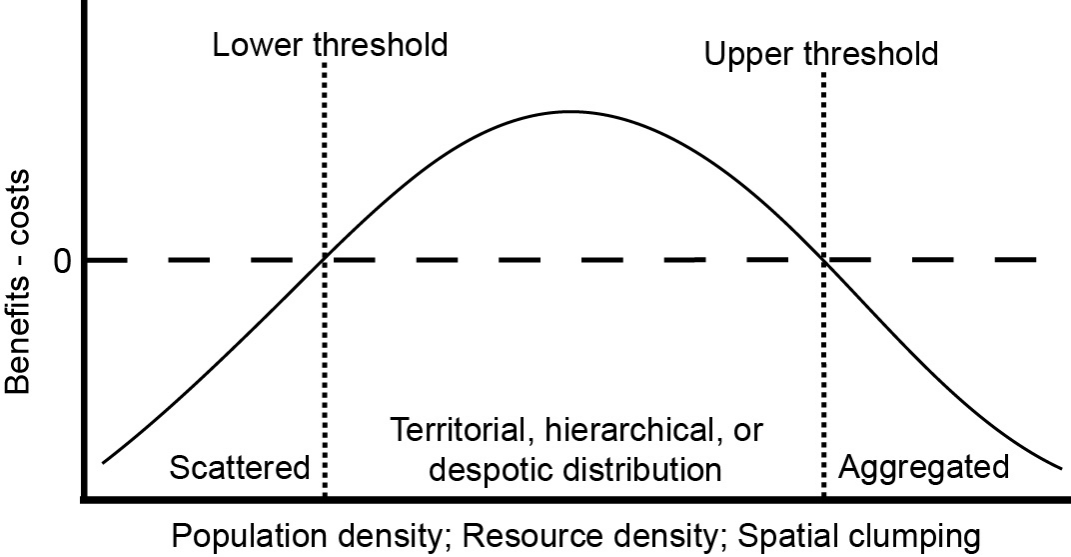
Resource defence theory. Ecological factors such as number and distribution of resources and competitors determine whether an individual guards a resource. Above the lower threshold, individuals aggressively defend space around a resource. Above the upper threshold, they cease to defend a resource. Graph based on [14] with the social structure that emerges under each condition added by the current authors. See text for detailed description.

If an individual is attached to a particular site while it is defending a resource, then the space defended is a territory. When resource density is low, the small benefit gained from defending a small amount of resource is not worth the costs incurred from driving away competitors, and individuals may be scattered as they travel long distances seeking resources (Fig.1, far left). As resource density increases, the benefits of maintaining exclusive use of the resource outweigh the costs of defending it, and an individual may behave aggressively, resulting in territoriality (Fig.1, lower threshold). As resources become so plentiful that they can be easily exploited by many, aggressive defence provides no benefit so it is no longer performed (Fig.1, upper threshold). The reaction to competitor density follows a similar pattern: At low competitor densities, individuals are scattered and most of the time there are no competitors nearby to guard against, so aggression is low (Fig.1, far left). When competitor density is higher, an individual may aggressively drive away competitors from a resource in order to maintain exclusive access to it (Fig.1, lower threshold). If space is limited and competitors are forced to remain within the territory as subordinates, then territoriality may take the form of a linear dominance hierarchy [17] or a despotic distribution in which all subordinates are of equal rank [18,19]. When competitors are very abundant, the costs of defence may exceed the benefits (Fig.1, upper threshold), so an individual may cease behaving aggressively [14] and may abandon a territory and join a group [20,21]. Such intra-individual plasticity in behaviour predicted from theory has been demonstrated empirically in diverse taxa [15]. Interestingly, territorial relationships may form without outright aggressive acts in many species, due to undisputed differences in competitive ability between individuals, ritualized behaviour, or prior social experience [8].

**Figure 2:**
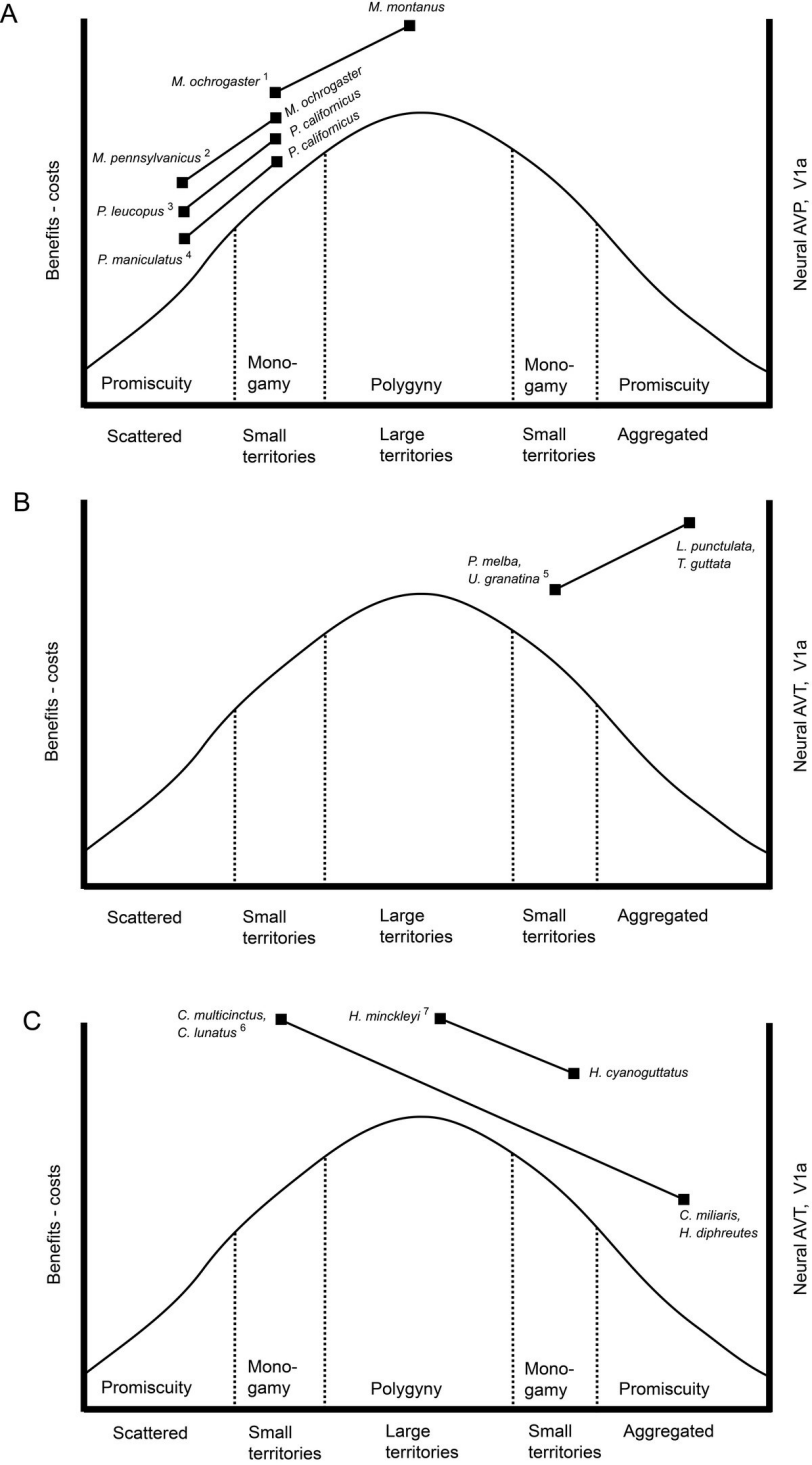
Studies that compared neural AVP, AVT, or V1a in males of different species. Comparisons are interpreted in the context of the inverted-U function that characterizes resource defense theory. Social organization is shown on the x-axis as a proxy for population density. The mating system that corresponds to each type of social organization is shown above the x-axis. For each inter-species study discussed in the text, each species is placed on the x-axis according to its species-typical social organization and mating system, and the levels of AVP, AVT, V1a in males for the two (or more) species compared are plotted on the y-axis relative to each other (without units). (*A*) In mammals, males of species with larger territories also exhibit greater septal circuit AVP and V1a. (*B*) In estrildid finches, mating system is dissociated from social organization, indicating that ecological factors other than population density or resource density shape mating system in these species. Studies all used monogamous species and AVT and V1a are not associated with territoriality or mating system. (*C*) In fishes, males of species with larger territories also have higher levels of neural AVT and V1a. ^1^V1a density in lateral septum [65,135,136], ^2^AVP-expressing axons in lateral septum [133], ^3^V1a density in lateral septum [52], ^4^V1a density in lateral septum [144], ^5^AVT neurons in BNST, baseline Fos levels in AVT neurons and social induction of Fos in AVT neurons, receptor binding density in lateral septum [61,147], ^6^Density of axons in ventral part of ventral telencephalon and size of cells in the gigantocellular POA that expressed AVT [95,149], ^7^V1a2 mRNA in the magnocellular and gigantocellular regions of the POA [150].

In many species, territorial behaviour has a strong influence on mating system, defined here as the time of, place of, and partner(s) mated, during mating [2,22]. Spatial and temporal distribution of resources and competitors may influence territorial behaviour, which then in turn influences mating system. From the male perspective during mating, females are resources and males are competitors. When population density is low, females are scarce or distributed broadly, and individuals may be scattered. Under such conditions, males may use a roaming tactic to find females and promiscuously maximize reproduction (e.g., African striped mouse, *Rhabdomys pumilio*[23]; Fig. 2, far left). If population density is higher and more competitor males are present, a male may establish a relationship with one female and defend a small territory, resulting in social monogamy (*sensu*[24]). This seems to be the case in several species of marine fishes ([25,26]; Fig. 2, center-left). At even higher population density, more females are available. Under these conditions, a male may be able to maintain a larger territory that includes more than one female, resulting in a polygynous mating system, as in the blue head wrasse, *Thalassoma bifasciatum* ([11]; Fig. 2, center). If population density is yet higher, the number of competing males and the costs of defending are high. A male may only be able to defend a small territory that includes only one female, resulting in monogamy (in some Central American cichlids competition for brood sites is so intense that cooperative defence between the male and female is required to successfully reproduce [27]; Fig. 2, center-right). If mating occurs in a population that is extremely dense, then there are abundant females with which to mate. Under these conditions, the costs of defending one female against the large number of male competitors may not be economical. A territorial male may not be able to exclude male competitors from a territory, and an aggregation may form (Fig. 2, far right). Aggregation is a second type of social organization within which individuals may mate promiscuously. In externally fertilizing species such as butterfly fishes, aggregated individuals may broadcast their gametes simultaneously to maximize fertilisation success [28].

It is important to note that other ecological factors can also influence social organization and mating system. Habitat use may influence distribution (animals aggregating in certain areas of the habitat due to unspecific physical factors like thermal environment, humidity, etc.; like bats in caves). The presence of hetero specific competitors or brood predators may restrict territory size or necessitate parental care [27,29], and high predation threat may cause individuals to aggregate [30]. For example, zebra finches, *Taeniopygia guttata*, form dense aggregations but are strongly monogamous [31]; most likely as a result of additional ecological factors that are beyond the scope of resource defence.

Here we present a perspective that extends the concept of intra- and inter-individual plasticity and reaction norm to and beyond the species level. Resource defence theory predicts intra-individual plasticity in behaviour. However, the same ecological factors that may lead to plastic behavioural responses in individuals may also lead to evolution in the tendency to behave territorially. This idea is consistent with recent conceptual arguments that suggest that behaviour may evolve by genetic accommodation, in which the degree of plasticity may not change, but the reaction norm of behavior may change in both effect size and in slope [32,33]. Population size fluctuates in many species [34], and likely attenuates selection for particular patterns of territorial behaviour. However, within a population, individuals vary both in their ability to plastically adjust behaviour according to local ecological conditions experienced at any given time and in their overall tendencies to behave territorially [35]. Therefore, long term changes in both the stability of ecological conditions and the overall typical state of those conditions could select individuals genetically predisposed to more or less readily adjust their behaviour to local conditions and also to tend to behave either less or more territorially in general. This would result in the evolution of differences in the typical patterns of social organization (e.g., scattered, small territories, large territories, aggregations) exhibited by different species [36]. Indeed, interspecies differences in territoriality are well documented [23]. Furthermore, studies that have found that differences between species in territorial behaviour and mating system may remain after ecological manipulation [37] indicate that in at least some species these differences are genetically selected and not simply plastic responses to local ecological conditions (see also [38]).

## AVP, AVT, V1A and behaviour

A substantial amount of research has sought to understand how certain neurochemicals (neurotransmitters or neuromodulators) influence behaviour. One family of neurochemicals, nonapeptides are nine-amino acid molecules that carry out numerous physiological functions, including the regulation of behaviour ([39,40]; Table 1). Oxytocin and its anamniote and avian/reptilian homologs (isotocin and mesotocin, respectively; [3,41] have numerous physiological roles, particularly in reproduction in females (e.g., inducing parturition and lactation in mammals), and have been shown to modulate social affiliation behaviour [42]. Another nonapeptide, arginine vasopressin (AVP), and its non-mammalian homolog arginine vasotocin (AVT), have many physiological functions and also influence social behaviour, primarily in males [3,41]. Associations of AVP (or AVT) and its V1a receptor subtype with a variety of social behaviour patterns including courtship, gregariousness, reproduction, aggression, and territoriality, have been established in diverse vertebrate classes [43,44].

**Table 1:** Nonapeptides and receptors that play a role in reproductive behaviour.

Molecule or structure	Description
Oxytocin	A nonapeptide associated with reproduction and social bonding. Thought to be most important in females.
Mesotocin	The bird/reptile homolog of oxytocin.
Isotocin	The anamniote homolog of oxytocin.
Arginine Vasopressin (AVP)	A nonapeptide associated with aggression, space use, and reproduction. Thought to be most important in males.
Arginine Vasotocin (AVT)	The non-mammalian homolog of AVP.
V1a	A cell membrane receptor for AVT and AVP that is commonly associated with social behaviour.
AVP circuit	A collection of neurons that project axons and deliver AVP or AVT to another set of neurons in a different region in the brain.

Note: Each nonapeptide is pleiotropic and has various physiological functions in other organ systems.

**Figure 3:**
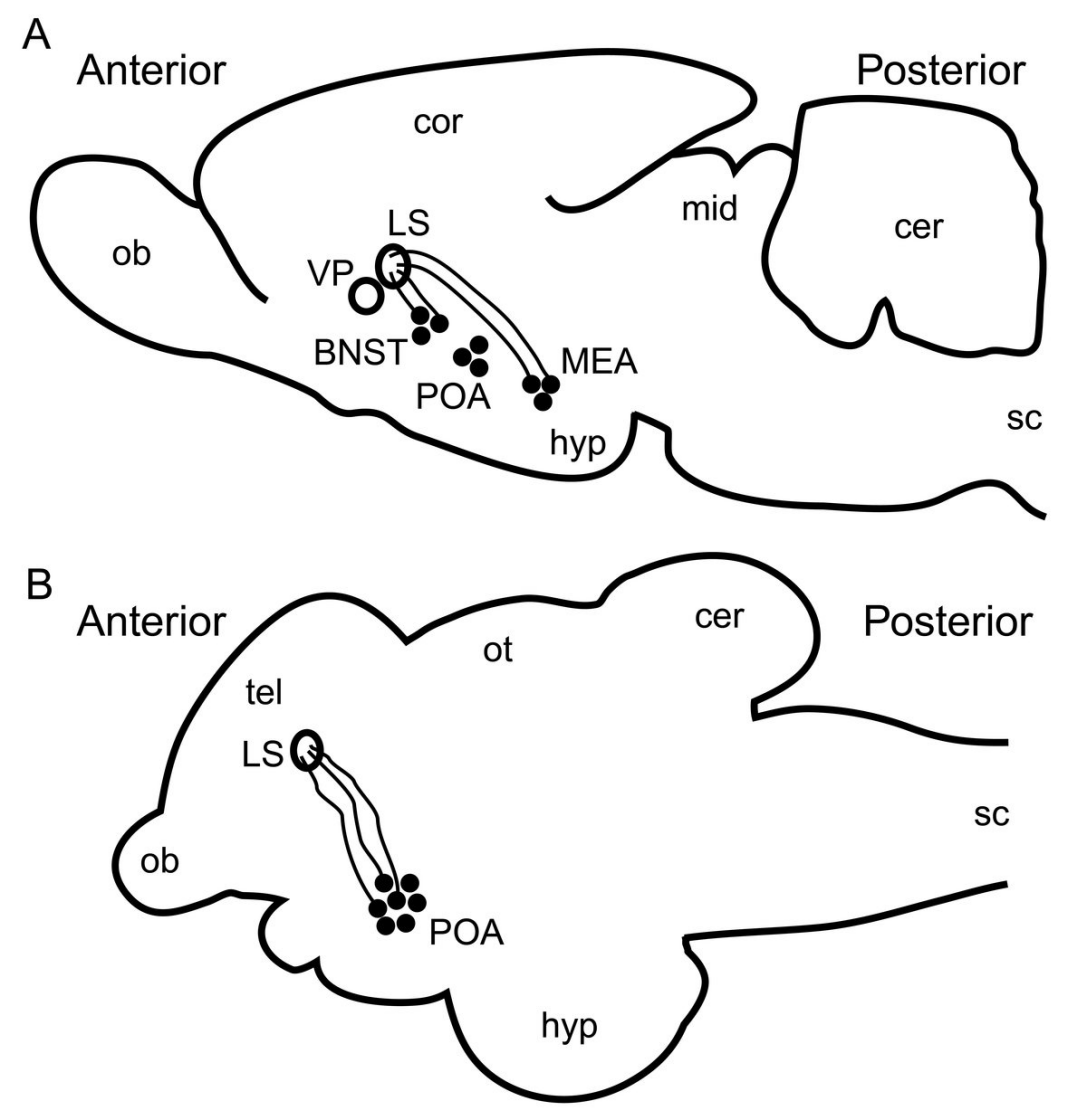
Lateral view of the brain of a typical (*A*) tetrapod (the mammalian prairie vole, *Microtus ochrogaster*) and (*B*) teleost fish (the cichlid *Astatotilapia burtoni*). Surface features are shown for orientation as lower case letters. Deeper structures of the AVP (tetrapod) or AVT (teleost) circuit associated with territorial behaviour are shown as upper case letters. The black dots represent AVP- (or AVT-) producing neuron cell bodies with axons projecting to the lateral septum. The anatomy of the septal AVT circuit in birds and reptiles is similar to that of the septal AVP circuit in mammals. Teleosts do not possess an anatomical lateral septum, but the ventral region of the ventral telencephalon is thought to be its homologous equivalent. BNST: basal nucleus of the stria terminalis; cer: cerebellum; cor: cerebral cortex; hyp: hypothalamus; LS: lateral septum; MEA: medial amygdala; mid: midbrain; ob: olfactory bulb; ot: optic tectum; POA: preoptic area; sc: spinal cord; tel: telencephalon; VP: ventral pallidum.

One of the best studied functions of AVP and V1a has involved a particular circuit in the brain that originates in the bed nucleus of the stria terminalis (BNST) and the medial amygdala and projects to the lateral septum (Table 2; [45-47]). AVP is produced in the cell bodies of neurons in the BNST and the medial amygdala. The axons of these neurons extend to the lateral septum where V1a receptors are found on their postsynaptic targets (Fig. 3A). In males of the monogamous prairie vole, *Microtus ochrogaster,* experiments using pharmacological methods and transgenic animals have demonstrated a causal relationship between AVP and V1a in these brain regions and behaviour patterns generally considered characteristic of monogamy, such as mate affiliation, nest defence, and paternal care for offspring [48-51]. The lateral septum also receives AVP from adjacent regions in a paracrine fashion. However, the simultaneous elevation of AVP in the BNST and V1a in the lateral septum when comparing sexes or species with different mating systems [52,53] suggest that this circuit plays an important role in all of these different behaviour patterns. An important question is how AVP and V1a expression can be associated with both affiliative behaviour and with aggressive defence of a territory, which appear to be very different forms of behaviour [54].

**Table 2:** Mammalian brain regions associated with mating system and their putative homologs in teleost fishes (see [80] for a detailed discussion).

Brain region	Description
Bed Nucleus of the Stria Terminalis (BNST)	Contains cell bodies of AVP- or AVT-producing neurons in tetrapods.
Medial amygdala	Contains cell bodies of AVP- or AVT-producing neurons in tetrapods.
Supracommissural nucleus of the ventral telencephalon (Vs)	Putative teleost homolog of the medial amygdala and BNST, but possesses no AVT-producing cell bodies.
Preoptic Area (POA)	A neuroendocrine integration centre located at the interface of the hypothalamus and telencephalon. Contains groups of AVP- or AVT-producing neuron cell bodies that project axons throughout the brain.
Paraventricular nucleus (parvocellular subdivision in teleosts)	Associated with stress and subordinate behaviour. In teleosts, part of the POA.
Supraoptic nucleus (magno-/gigantocellular subdivision in teleosts)	Associated with aggression and reproductive behaviour. In teleosts, part of the POA.
Lateral septum (LS)	In tetrapods, a collection of neurons in the medial forebrain lying generally anterior to the anterior commissure. Receives axons of AVP- or AVT-producing neurons. Associated with several types of social behaviour.
Ventral nucleus of the ventral telencephalon (Vv)	Putative teleost homolog of the lateral septum.
Ventral pallidum (VP)	A brain region immediately ventral to the lateral septum in tetrapods. Receives axons of AVP- or AVT-producing neurons. Implicated in social pair-bonding in rodents.

One possible explanation for the septal AVP-V1a circuit's stimulatory effect on both affiliation and aggression could be that it more generally enhances social cognition and in doing so stimulates the formation all types of social relationships [55]. Here we define social cognition specifically as the acquisition and retention of social information [56]. Territoriality would seem to require establishing relationships with other individuals (albeit a confrontational context) and the recognition of individuals upon a subsequent encounter. Social cognition may thus be necessary to form and maintain relationships with both mates and offspring and with competitors (such as familiar neighbours or “dear enemies”) when maintaining a territory, compared with simply roaming over a broad home range [44,57-59]. Additionally, holders of large territories are probably continually establishing new relationships as neighbouring individuals change, compared to holders of small territories who have fewer neighbours, or compared to roamers who may encounter many individuals but never form a relationship. Therefore, up-regulation of a neural circuit that stimulates social cognition, specifically the establishment and maintenance of social relationships in specific surroundings, could facilitate both affiliative and aggressive behaviour, depending on social context.

In the sections below, we review studies that have measured aspects of neural AVP, AVT, and/or V1a. The studies are of two general designs: they either measure and compare neural AVP, AVT, and/or V1a between different behavioural phenotypes or they manipulate neural AVP, AVT, and/or V1a and measure the resulting behaviour. Studies that measured neural AVP, AVT, and/or V1a have done so using a variety of different methods. Many studies used immunohistochemistry (e.g., [60]), a laboratory technique used to anatomically visualize the location of a specific protein by using a specific antibody that binds to it. The antibody is visible with a fluorescence microscope when it is bound to a special light-emitting molecule. These studies typically then compare the sizes or numbers of AVP or AVT-immune-reactive (AVP-ir) cells or density of staining within the cells. Some studies measure *c-Fos* expression in a similar way in addition to the AVP- or AVT-ir (e.g., [61]). Immediate early genes, such as *c-Fos*, are rapidly and transiently upregulated following an external stimulus. Therefore, increased expression of both AVP or AVT and *c-Fos* suggest that an AVP- or AVT-expressing neuron is increasing activity in response to a stimulus. Other studies have measured AVP or AVT mRNA. *In situ* hybridization binds mRNA using a radioactively labelled complementary strand of DNA or RNA (e.g., [62]). Quantitative PCR measures AVP or AVT mRNA by binding it with a light-emitting molecule (e.g., [63]). Finally, a few studies have measured AVT directly using high-performance liquid chromatography; a new application of this method (e.g., [64]). V1a receptors have been identified using autoradiography: binding to a radioactively labelled ligand (e.g., [65]). Many studies that have manipulated neural AVP, AVT, and/or V1a have directly applied AVP, AVT, or V1a antagonist. This has been done by either infusing AVP or V1a antagonist into one of the brain ventricles, (e.g., [66]) or directly injecting AVP or AVT into a specific brain region or into the intraperitoneal cavity (e.g., [67]). A few studies have “knocked down” AVT production in a particular brain region by injecting antisense oligonucleotides that bind to the target mRNA and prevent it from being translated [68].

## Intra-species variation in AVP, AVT, V1a and territoriality

A substantial number of studies have examined neural AVP, AVT, and V1a within and among individuals of a particular species. In this section, we review those studies to asses if neural AVP, AVT, or V1a is closely associated with either aggression or territoriality. An association with aggression would indicate a direct relationship between the molecular pathway and aggression. An association with territoriality would suggest that the pathway has a cognitive function, which would better explain the perplexing diversity of behaviours that have been associated with AVP, AVT, and V1a in previous studies. Furthermore, if neural AVP, AVT, or V1a is associated with territoriality within species, then it may also be possible for neural AVP, AVT, and V1a to also be associated with territoriality between species.

### Intra-species variation in mammals

Many studies have demonstrated a relationship between neural AVP and V1a and aggression in rodents [44,69,70]. Most studies that have investigated the role of AVP or V1a in aggression have relied on resident-intruder tests, which alone are not sufficient to distinguish increases in pure aggressive behaviour from aggression performed specifically as a form of territory defence. However, one study specifically found that in one particular species, the aggression stimulated by neural AVP and V1a is performed not simply to injure or drive away another individual, but to establish a social relationship in the context of a particular place (territory). Bester-Meredith et al. [66] cleverly examined the effects of intracerebroventricularly (ICV) infused AVP and V1a antagonist on aggression in both a resident-intruder test and a neutral arena test. In the monogamous, territorial California mouse, *Peromyscus californicus*, the antagonist lengthened attack latencies in the resident-intruder test, but had no effect in the neutral arena test. In the promiscuous white-footed mouse, *P. leucopus*, which does not typically maintain exclusive territories (see Inter-Species Variation in Mammals section below) the antagonist did not alter attack latencies in either test. These results indicate that neural V1a functions specifically in territorial behaviour in California mice but is not a mechanism that strictly increases aggression in either of these species [66].

Additional evidence that the function of neural AVP and V1a extends beyond simply increasing aggression and instead functions specifically to establish social relationships in the context of territoriality can be found in Syrian hamsters, *Mesocricetus auratus*. Males of this species rub secretions of special glands onto objects in their territories in response to cues from other male hamsters, an activity known as flank marking. AVP injection into the medial preoptic area / anterior hypothalamus, BNST, medial amygdala, and lateral septum of males stimulates flank marking in the absence of other hamsters, and is mediated by V1a in the lateral septum [71-74].

One study has examined the role of septal V1a in natural intra-species variation in space use in male prairie voles. Prairie vole males are typically considered monogamous and territorial, but they may also exhibit a wandering tactic in which they are not site attached but roam and mate opportunistically with females they encounter [75-78]. Within-species comparisons among individual males failed to find a significant difference in septal V1a between territorial males and wandering males [79].

### Intra-species variation in birds

The anatomy of the septal AVT circuit in birds is similar to the septal AVP circuit in mammals (see Figure 3 in [80]). In birds, some studies have supported a relationship between neural AVT and V1a and aggression and territoriality and some have not. In a well-studied polymorphism in white-throated sparrows, *Zonotrichia albicollis*, white-striped males defend their territories more vigorously and intrude into other territories for extra-pair copulations more often than do tan-striped males, and they have more AVT-ir (immune-reactive) expression in the medial portion of the BNST and in the ventrolateral subdivision of the caudal lateral septum [60]. In this species, neural AVT and aggression are clearly associated with a situation in which increased social cognition would benefit a territory-holding male in identifying and remembering nearby males and females.

The relationship between neural AVT and V1a and behaviour is more complex in estrildid finches. In the highly social and monogamous zebrafinch, aggression decreases with time when individuals are group-housed, but is higher in paired compared with unpaired males. Intracerebroventricular AVT injection increases, and V1a antagonist decreases, aggression during competition to court [81,82]. Furthermore, increases in AVT-Fos are observed in a mate competition paradigm if subjects are allowed to court, but not if they are aggressively subjugated [61]. Kabelik et al. [83] injected a mixture of V1 antagonists into the lateral ventricle and observed a decrease in aggression in unpaired males on the first day of group formation, when aggression was performed as competition over mates. However, paired males increased aggression after the same treatment. In territorial species, the field sparrow, *Spizella pusilla* [84] and the violet-eared waxbill, *Uraeginthus granatina* [85] intraseptal AVT infusions inhibit resident-intruder aggression. On the other hand, bilateral knockdown of AVT production in the BNST using antisense oligonucleotides and intraseptal infusions of V1a antagonist in male zebra finches reduced gregariousness: the tendency to associate with a large group (n=10) over a small group (n=2) [68]. The antisense oligonucleotides also decreased exploration behavior in a novel environment [68]. In the moderately gregarious Angolan blue waxbill (*Uraeginthus angolensis*), bilateral antisense knockdown of AVT production in the BNST reduced social contact, but not gregariousness, especially in males [86]. Thus, in estrildid finches, AVT and V1a of the septal circuit variously promotes aggression, social contact, or gregariousness, suggesting a general social cognition function that varies depending on social context and on species.

### Intra-species variation in reptiles and amphibians

The septal AVT circuit in reptiles and amphibians is anatomically similar to the septal AVP circuit in mammals (see Figure 3 in [80]). Neural AVT influences reproductive and aggressive behaviour in reptiles and amphibians [87,88], and many species in these taxa exhibit territorial behaviour and diverse mating systems [89-91]. Neural AVT is well known to stimulate mate calling in frogs, but two studies specifically observed results pertinent to the role of AVT in territorial behaviour. In staged encounters involving the gray tree frog, *Hyla versicolor*, intraperitoneal (IP) AVT injection increased an intruder male's ability to acquire calling sites from resident males without physical aggression [67]. Similarly, after IP injection of AVT in the common coquí, *Eleutherodactylus coqui*, satellite males left the territories of other males and formed their own new territory and began calling [92]. In contrast to this pattern of association between AVT and territoriality, Marler et al. [93] found that satellite males possessed significantly more AVT-ir, in terms of both density of staining within cells and in cell size, in the nucleus accumbens of the brain than did calling males. These studies suggest a role for neural AVT in territoriality in amphibians, but the specific brain regions involved remain unclear.

### Intra-species variation in teleost fishes

In teleost fishes, neural AVT and V1a expression have often been associated with aggression and territoriality, although several studies have found an inverse relationship (reviewed by [94]). Some studies in teleosts have observed an association between territorial behaviour and measures of AVT and V1a in distinct forebrain regions that have putative mammalian homologs (Fig. 3B; Table 2; [6]). In teleosts, AVT-expressing neuron cell bodies are located only in the parvocellular, magnocellular, and gigantocellular portions of the preoptic area (POA) and (to a lesser extent) the lateral tuberal nucleus of the hypothalamus [95-98]. Several studies [96,98-101] have found that aggressive males of a particular species have larger or more numerous AVT-ir cell bodies than do non-aggressive males in the gigantocellular portion of the POA, and sometimes non-aggressive males have more in the parvocellular portion, the putative teleost-homolog of the paraventricular nucleus [3]. In many of these studies, the males compared were specifically identified as being either associated with a specific site or not, so the aggression performed comprised territoriality [96,98,100,101]. One study found larger parvocellular and magnocellular AVT-ir neurons in males of a less aggressive population, but the large size may have been due to due greater function in osmoregulation [102]. Another study found fewer AVT neurons in the magnocellular layer of the POA in territorial individuals after controlling for body size [103], although the pattern was observed in juveniles of a species that undergoes a natural social status-based male to female sex change (clown anemonefish, *Amphiprion ocellaris*), so reduced AVT might have been indicative of impending female differentiation. Other studies measured AVT mRNA or AVT directly. These have often found higher levels of AVT in the brains of territorial individuals (e.g., [64,96,104-106]) although at least one study found no relationship [107].

Manipulations of neural AVT and V1a have also often produced results consistent with a role in territoriality. Intraperitoneal injection with a nonapeptide receptor antagonist delayed the initiation of both aggressive and affiliative behaviour in male convict cichlids, *Amatitlania nigrofasciata*, when introduced to a potential mate and competitors [108]. In territorial male Beaugregory damselfish, *Stegastes leucostictus*, IP-AVT injection resulted in an increase in aggressive behaviour toward intruders in a dose-dependent manner, and injection with a V1a receptor antagonist reduced aggressive behaviour [109]. In other species, neural AVT enhancement inhibited aggression and dominance. Juvenile rainbow trout, *Oncorhynchus mykiss*, receiving AVT injected into the 3^rd^ ventricle became subordinate, and those that received V1a antagonist tended to become dominant [110]. In the weakly electric brown ghost knife fish, *Apteronotus leptorhynchus*, IP-AVT injections increased courtship chirps but inhibited agonistic chirps in males [111]. Intraperitoneal injection of AVT has also been found to reduce rates of aggression in Amargosa River pupfish, *Cyprinodon nevadensis amargosae* [112]. In one study, such a negative relationship was observed in the zebrafish, *Danio rerio* [106], and was in contrast to the positive association between magnocellular AVT neuron size and aggression previously observed in the same species [99].

**Table 3:** A general classification of mating system variation with an emphasis on territory defence if it sometimes occurs in a particular mating system [2].

Mating system	Description
Promiscuity	Individuals mate with multiple members of the opposite sex, sometimes indiscriminately. Typically there is no pair bonding. Individual home ranges overlap with those of same sex and those of the opposite sex [171,172].
Polygamy	Ongoing mating with a group of multiple mates. Can be subdivided into polygyny and polyandry, depending on which sex is polygamous (2,131,171,172).
Polygyny	A type of polygamy in which one male mates with multiple females. In territorial polygynous systems, each male holds a territory from which other breeding males are typically excluded, and which contains the territories or home ranges of females. The males’ territories do not overlap with the territories of other males, and the female territories only overlap with the territory of a single male. Males may mate with the same females in successive mating attempts [171]. Social relationships apparently exist between a territorial male and each female, but they do not spend as much time in coordinated activities as do monogamous pairs. Relatively common [2,172].
Polyandry	A type of polygamy in which one female mates with multiple males for successive breeding attempts. Relatively rare compared to polygyny [2,171].
Polygynandry	Members of each sex mate with multiple partners. Not tied to resource defence. Often used instead of “Promiscuity” to distinguish species that do not mate indiscriminately, but may or may not involve ongoing relationships (see “Lek,” below).
Lek	A type of polygyny or polygynandry[173] in which males form aggregations of small display territories and compete for dominant status during breeding season and females choose among them. Males do not contribute resources or parental care; females visit briefly only to have their eggs fertilized. There are no social bonds [171,174].
Monogamy	A female and a male form a social bond, often mutually defend a territory, and often cooperate to care for their offspring. Environmental constraints prevent either sex from monopolizing more than one member of the opposite sex. Adults remain in close proximity to each other. May be serial, with partners changing with each breeding attempt, or long-term [171,172].
	

Studies of fish species with alternative male mating tactics have provided opportunities to examine intra-species variation in the role of neural AVT and V1a in territoriality, and have produced contrasting results. In male bluehead wrasses, *Thalassoma bifasciatum*, territorial males did not have more numerous AVT-ir neurons, but AVT mRNA within magnocellular neurons in territorial males tended to be greater than in the smaller, reproductive but non-territorial male phenotype [113]. Furthermore, qPCR revealed in both whole-brain and hypothalamus samples that V1a2 (one of the two V1a paralogs present in teleosts [114]) was also higher in territorial males [63]. After IP injection with AVT or a V1a receptor antagonist in males, AVT generally increased territorial behaviour and V1a antagonist decreased territorial behaviour, which was not limited to aggression, but also involved remaining at one site instead of roaming, refraining from feeding, and increasing inspections of conspecifics [115]. Furthermore, AVT was necessary to assume territorial status in both non-territorial males and sex-changing females [116]. In contrast, in the plainfin midshipman, *Porichthys notatus*, dominant, territorial males have AVT-ir neurons in the entire POA that are larger than in smaller, non-territorial sneaking males, which has been interpreted to be an insignificant effect of larger body size. However, non-territorial males have more AVT-ir neurons per body mass [117]. Similarly, if corrected for body mass, the smaller non-nesting males of the Azorean rock-pool blenny, *Parablennius parvicornis,* have significantly more AVT-ir cells in the entire POA than do the large nesting males [118]. One study was able to distinguish whether neural AVT was more strongly associated with territoriality or courtship toward females [62]. In the peacock blenny, *Salaria pavo*, territorial males do not perform courtship behaviour [119]. In the entire POA, immunocytochemistry found that there were no differences in body size-corrected AVT-ir cell size or numbers between territorial morphs and sneaking morphs. However, AVT mRNA expression on a per-cell basis using *in situ* hybridization was higher in sneaker males than in nest-holders, indicating that AVT in this species is associated with courtship behaviour more than it is with territoriality. This is consistent with another study in which IP injections of AVT induced the expression of courtship behaviour in sneakers but not in territorial males [120]. Therefore, in some species, neural AVT and V1a are associated with territoriality, but in others they are more strongly associated with courtship.

### Intra-species variation in invertebrates

Little is known about the mechanisms that might underlie aggression and territoriality in invertebrates. In arthropods in particular, many species are territorial [121,122], and aggressive defence of resources is at least partially mediated by a known molecular mechanism (juvenile hormone) in some species [123]. Distinct nonapeptides are found in different invertebrate taxa [41], including some arthropods such as ants [124,125], although no such molecules have been found in eusocial honeybees [126]. Oxytocin/vasopressin homologs induce reproductive movements in leeches, worms, and snails [127,128] and affect learning and memory in cuttlefish [129], but there is at this time no evidence that nonapeptides play a role in territoriality in invertebrates. Research into the neuromolecular mechanisms regulating territoriality in invertebrates is a promising avenue for understanding the evolution of social behaviour.

## Interspecies variation in AVP, AVT, V1a and territoriality

Below we review studies that have compared aspects of neural AVP, AVT, or V1a in two or more species that differ in aggression, territoriality, and mating systems and interpret them in the context of resource defence theory. On an inverted-U resource defence function we plot relative expression of neural AVP, AVT, and V1a, using social organization (e.g, scattered, large territories, aggregations) as a proxy for resource density and competitor density, then we evaluate congruency between patterns of neural expression and the species-typical level of territorial behavior (no territories, small territories, large territories) (Fig. 2). Just as our analysis assumes that species-typical patterns of social organization are selected in response to species-typical ecological conditions and population densities, it also assumes that neural AVP, AVT, and V1a are consistent enough within species to persist under the artificial social conditions elicited by a laboratory environment. Indeed, the statistically significant differences between species reported in numerous published studies (see below) suggest that neural expression within a species is consistent enough to persist under artificial socio-ecological conditions (*sensu* [61]). Many of the studies discussed below are two-species comparisons, which are limited in their ability to allow conclusions about the evolution of traits [130]. However, multiple observations of a particular pattern more strongly indicate that the pattern is a result of evolutionary change and not merely a random difference between species [130].

### Inter-species variation in mammals

In studies that compared males of two mammal species, expression patterns in the septal vasopressinergic circuit mirrors territory size. Prairie voles are typically socially monogamous (but see discussion above in the section Intra-Species Variation in Mammals), and male-female pairs usually share a common nest and home range, from which males exclude other males, i.e., a territory [131]. Meadow voles, *M. pennsylvanicus*, in contrast, are promiscuous and the males are not territorial. Females have small home ranges and distinct territories, but males’ home ranges are large and overlap considerably, and males frequently enter the territories of estrous females [132]. The number of male home ranges that overlap with female home ranges is similar to the number of female home ranges that overlap with male home ranges. Wang [133] found that male prairie voles had a higher density of AVP-expressing axons in the lateral septum than did male meadow voles (Fig. 2A). Interestingly, male montane voles, *M. montanus*, which are polygynous and maintain exclusive, long-term territories that are even larger than those of male prairie voles and which encompass several smaller female territories [134], show even greater expression in the septal vasopressinergic circuit: Comparisons of receptor V1a densities in the lateral septum between prairie voles and montane voles have found higher levels in *M. montanus* [65,135,136]. Therefore, expression of both AVP and V1a in the lateral septum is associated with male territory size in *Microtus* voles (Fig. 2A).

The vasopressinergic circuit is also associated with territoriality in mice of the genus *Peromyscus*. These species show variation in territorial behaviour similar to that found in *Microtus* voles. Mated pairs of the monogamous California mice are strongly territorial, maintaining exclusive home ranges [137]. Males spend more time providing parental care and attack opponents more rapidly than do males of the promiscuous white footed mice [52], which cease to nest with females after the litters are born and provide no paternal care [138]. White footed mice may sometimes aggressively defend their home range as a territory, but only at high densities, and even then there is some overlap among the males’ home ranges [139,140]. In fact, females’ home ranges are more strictly exclusive of same-sex individuals than are males’ home ranges [139]. Low densities are more common[141], and although under these conditions males’ home ranges are exclusive of each other, resident males frequently leave their home ranges to roam in search of females [142], and contact between males is frequent but usually does not involve aggression [141]. Importantly, male California mice show a larger area of neuron cell bodies and axons stained for AVP in the BNST and more V1a receptors in the lateral septum than do male white footed mice([52]; Fig. 2A). Similarly, home ranges of adult deer mice, *P. maniculatus*, overlap broadly with those of members of the same and opposite sexes, and both males and females are promiscuous [143]. Insel et al. [144] found that male California mice have more V1a receptors in the lateral septum than do male deer mice. In sum, in comparative studies of both *Microtus* voles and *Peromyscus* mice, the species that exhibits the greatest exclusive use of space also shows the highest expression in the septal vasopressinergic circuit (Fig. 2A).

Three comparative studies that did not find a positive relationship between neural AVP or V1a and territorial behaviour should be mentioned. First, Wang et al. [145] found that males of the monogamous pine vole, *M. pinetorum*, had larger areas covered by AVP-expressing axons in the lateral septum than did prairie voles, meadow voles, or montane voles. However, for unknown reasons this study also failed to observe any of the other species differences in expression that had repeatedly been reported in previous studies. Next, Insel et al. [65] found that pine voles had less V1a in the lateral septum compared to the promiscuous meadow voles. However, that study did not analyse males and females separately, which likely obscured any relationship between V1a and territory size that might exist only in males. Finally, Turner et al. [146] failed to find an association between V1a density in the lateral septum and mating system in males of *Peromyscus* spp. However, their analysis likely suffered from a lack of power due to small sample sizes (only two males of each of five species).

### Inter-species variation in birds

In birds, studies have compared neural AVT and V1a in gregarious (aggregating) vs. territorial estrildid finch species. All species of this group are monogamous, so mating system is dissociated from social organisation [3]. Although the septal vasotocinergic circuit has been associated with aggression in some contexts in some species (see Intra-Species Variation in Birds section, above), across species it is more strongly associated with grouping behaviour [3]. Specifically, the gregarious zebra finch and spice finch (*Lonchura punctulata*) show more AVT-expressing neurons in the BNST, and higher baseline Fos levels and greater social induction of Fos in those AVT neurons than do the territorial species, the Melba finch (*Pytilia melba*) and the violet-eared waxbill (*Uraeginthus granatina*). The moderately gregarious species, the “Angolan” blue waxbill (*U. angolensis*), typically exhibits intermediate values [61]. Similarly, an antagonist known to bind to mammalian V1a was found to bind in several septal areas typically in higher densities in the zebra finch and spice finch than in the Melba finch and the violet-eared waxbill [147]. This pattern seemingly contrasts with the association of septal AVP and V1a and territoriality described above for cricetid rodents (Fig. 2B). However, Goodson & Wang [61] introduced the idea of social valence to explain their findings: septal-circuit AVT and V1a across species is associated with positive interactions and inversely associated with negative interactions. From this perspective, a competitive social interaction and social attraction may both be positive, whereas experiencing aggressive subjugation would be a negative interaction (see also [43,148] for further discussion of the “social valence” concept).

### Inter-species variation in teleost fishes

Similar to the situation described above for cricetid rodents, expression in neural AVT and V1a in the brains of males varies across fish species with characteristically different male territory sizes (in the brain region homologous to the mammalian lateral septum: the ventral nucleus of the ventral telencephalon [area Vv]; see [80]). The coral reef-dwelling butterflyfishes, Chaetodontidae, provide a compelling example. The multiband butterflyfish, *Chaetodon multicinctus,* is a monogamous species in which the male and female work together to defend feeding territories against competitors [28]. This species does not use the territories for breeding, but the male provides territory defence and thus food to the female, and the female provides reproductive opportunity to the male [26]. The milletseed butterflyfish, *C. miliaris*, in contrast, is a pelagic species that schools and breeds promiscuously in groups [28]. Dewan et al. [95] found that males of the territorial multiband butterflyfish had larger AVT-expressing neuron cell bodies (in the gigantocellular and magnocellular regions of the POA) and higher AVT-expressing axon densities (in area Vv) than did the non-territorial milletseed butterflyfish (Fig. 2C). Dewan et al. later [149] compared males of seven butterflyfish species and confirmed quantitatively that the number and size of AVT-ir cells in the gigantocellular preoptic cell group and the density of AVT-ir varicosities in the Vv were generally greater in those species with mating systems characterised by male territories.

A recent study by Oldfield et al. [150] compared males of two heroine cichlid species with different mating systems: the Cuatro Ciénegas cichlid, *Herichthys minckleyi*, in which males are polygynous and maintain large territories, and the Rio Grande cichlid, *H. cyanoguttatus*, in which males form monogamous pair-bonds with females and defend small, temporary nesting territories [38]. These authors found that neural expression of V1a2 in males was associated with territory size. Specifically, in gross-dissected tissue containing the gigantocellular and magnocellular regions of the POA, V1a2 mRNA in territorial male Cuatro Ciénegas cichlids was higher than in territorial male Rio Grande cichlids and non-reproductive males of either species [150]. Even though anatomical resolution was limited in this study, the pattern of higher neural V1a2 expression in males in species in which males have larger territories is similar to that observed by Dewan et al. [95,149], and suggests a similar, and possibly conserved, mechanism in different teleost families (Fig. 2C). Furthermore, this neural mechanism underlying variation in territory sizeis at least in part conserved between mammals and teleost fishes even though patterns of territoriality have clearly evolved independently [6,38].

## Conclusion

The results of our analysis indicate that neural vasopressinergic and vasotocinergic action facilitates territoriality, which in turn may shape mating systems. We found that, within species, neural AVP, AVT, and V1a are often associated with territorial behaviour. Similarly, among species, we found that neural AVP, AVT, and V1a are associated with territorial behaviour in a pattern consistent with resource defence theory. This suggests that ecological pressures may, through natural selection, drive up- or down-regulation of expression in a particular neural circuit that stimulates territorial behaviour, resulting in the evolution of differences in reaction norms between species. Because territorial behaviour can influence the acquisition of mates, then such a neural circuit may also influence the evolution of mating systems.

One possible reason that neural AVP, AVT, or V1a expression might be higher in territorial individuals may be because it directly stimulates outright aggressive behaviour (reviewed by [70]). However, several studies indicate that AVP, AVT, and V1a do not necessarily stimulate aggressive behaviour but instead initiate an aggressive disposition, which might be beneficial during the establishment of a social relationship. Although male prairie voles ICV-infused with a V1a antagonist failed to develop a typical increase in aggressive behaviour after co-habitation with a female, aggression was not reduced in males that had previously-established pair-bonds with females [48]. Decreases in aggression caused by nonapeptide antagonists in birds and fishes were also restricted to occurring during establishment of a relationship but not after a relationship had formed [83,108]. Furthermore, although Oldfield et al. [150] found neural V1a2 expression to be associated with territory size in two species of cichlid fishes, raw numbers of aggressive acts compared between territorial males of each species were negatively associated with V1a2 [38]. Therefore, although neural AVP, AVT, and V1a may stimulate aggression in mammals, birds, and fishes, it seems to do so primarily during the formation of social relationships and not necessarily in other contexts.

The role of neural AVP, AVT, and V1a in the formation of social relationships could be related to its known role in general social cognition. AVP and V1a in the lateral septum are well known to facilitate social memory [55]. For example, in rats, V1a agonists injected into the lateral septum facilitate, but V1a antagonists and AVP anti-sense DNA inhibit, the memory of same-sex conspecifics and familiar juveniles [151]. The association described above between AVT and V1a in the septal circuit and gregariousness in finches is consistent with this idea – the tendency of gregarious animals to approach other individuals and to choose to associate with large groups over small groups is similar to the tendency of animals with large territories (compared to animals with small territories or roaming-tactic species) to establish social relationships [152]. Thompson & Walton [153] found that AVT ICV-administered to highly social individual male goldfish, *Carassius auratus*, inhibited social approach toward same-sex conspecifics, but treatment with an AVT receptor antagonist stimulated social approach [153], but this mechanism was later found to be mediated by a different circuit in the hindbrain [154]. Therefore, neural AVP, AVT, and V1a, at least in one circuit in the forebrain, seems to be responsible for generally positive-valence behaviour important for establishing social relationships, which may include aggression, but also territoriality (independent of aggression), and gregariousness.

Ophir [5] proposed a two-axis interaction between social bonding and social spacing that determines overall social behaviour, such that seemingly contradictory aspects of social behaviour may in fact arise from the coordinated action of multiple vasopressinergic circuits in the brain (see also [3,96]). The finding that V1a receptors are widely distributed throughout the vertebrate brain [155-158] and that this expression pattern is highly conserved across vertebrates [159], is consistent with the notion that AVP (or AVT) and V1a expression in different brain regions likely mediates different behavioural processes such as social bonding and territoriality in a manner that is similar across lineages [3,6,94,96,148,160]. The septal vasopressinergic or vasotocinergic circuit could variably stimulate additional downstream circuits, each of which are more directly responsible for either affiliative or aggressive behaviour. Alternatively, there may be subdivisions within the septal vasopressinergic or vasotocinergic circuit that are differentially responsible for stimulating different forms of social behaviour [161].

We also found that, at least in mammals and fishes, differences between species’ mating systems are consistent with differences in neural AVP, AVT, and V1a expression. Despite the apparent connection with monogamy in prairie voles [48], across different vole species the association between the septal vasopressinergic circuit and monogamy has not appeared to be very robust: the higher abundance of V1a in the lateral septum in *M. montanus* than in *M. ochrogaster* [65,135,136] had previously been interpreted to be inconsistent with the idea that the septal vasopressinergic circuit was associated with mating system across species [3,5]. To reconcile these disparate results, Goodson and Bass [162] proposed the idea that V1a expression in the lateral septum of cricetid rodents is consistent with the patterns of space use observed in different mating systems (i.e., home range size and overlap), but not with pair bonding. The current work expands this idea by showing how stimulation of a neural vasopressinergic or vasotocinergiccircuit could enhance social cognition and the formation of social relationships, thereby facilitating territorial ownership and influencing mating systems in those species in which males may defend resources in order to mate. It is important to note, however, that these factors do not necessarily interact in this way to determine mating system in all species. In some species, other ecological factors (such as high predation threat or need for parental care) may play a key role in shaping social organization and mating system. In estrildid finches, overall social organization is dissociated from mating system. Species may exhibit territorial or aggregating social organizations, but all are monogamous. Thus it is not surprising that septal circuit AVT and V1a do not predict territoriality or mating system in these species.

Although the septal vasopressinergic or vasotocinergic circuit has clearly been found to be responsible for stimulating affiliative and gregarious behaviour, pair bonding in male *Microtus* spp. is likely mediated not by the lateral septum but by the ventral pallidum, a region immediately ventral to the lateral septum [163]. Although males of the polygynous *M. montanus* have higher V1a densities in the lateral septum than do monogamous male *M. ochrogaster*, the latter have higher densities of V1a in the ventral pallidum [164]. Blocking the binding sites of V1a in the ventral pallidum prevented mating-induced preference for a particular female partner in male *M. ochrogaster* [163], and experimental induction of V1a receptor expression in the ventral pallidum of *M. pennsylvanicus* dramatically increased partner-preference in this normally promiscuous species [164]. Young & Wang [163] proposed that pair bonding in *M. ochrogaster* is caused by an interaction between AVP in the ventral pallidum that causes partner preference and AVP in the lateral septum that causes partner recognition (in combination with dopamine in the nucleus accumbens). A teleost fish homolog of the mammalian ventral pallidum has not yet been conclusively identified [80], but a recent developmental and neurochemical study suggests that it may be the caudal portion of area Vv [165]. Additionally, recent evidence has found that monogamous male prairie voles expressed higher OT receptor density in the nucleus accumbens than did non-monogamous males [166], a pattern proposed previously to be important to elicit mating-induced partner preference in females [163]. It will thus be fruitful to examine whether a similar pair-bonding circuit may be present in other vertebrate taxa, and whether it involves AVP, AVT, or OT or isotocin [167].

To fully understand the mechanisms underlying different mating systems, more data are needed from females. Female behaviour plays an important role in shaping mating systems. In many species females maintain territories that may or may not be congruent with male territories (see examples provided above for cricetid rodents), and mating systems emerge from conflict between male and female interests [168]. Unfortunately, we have not found published data on the relationship between nonapeptides and territorial behaviour in females. It seems likely that a neural mechanism underlying space use in females might involve OT, which has been found to vary with social structure in tuco-tucos [169]. In fact, OT has recently been found to activate V1a receptor and weakly stimulate flank marking in male Syrian hamsters, so it may play a role in shaping mating systems in males as well [170].

In sum, we have identified an association between a proximate-level neuro-endocrine mechanism and an ultimate-level behaviour pattern that has a direct effect on the social organization of populations. Specifically, neural AVP, AVT, and V1a are associated with territorial behaviour in males, and the most parsimonious explanation for the role of AVP, AVT, and V1a in territorial behaviour is to stimulate social cognition, not necessarily aggression. Furthermore, we have applied the concept of intra- and inter-individual plasticity and reaction norm to evolutionary patterns at and above the species level. Because mating in some species involves territorial behaviour, neural AVP, AVT, and V1a also seem to mediate mating system in both mammals and teleost fishes, but not in the bird species studied to date. Territorial behaviour is most likely one key trait driving resource defence-based mating systems, whereas mating systems are emergent properties arising from the presence or absence of territorial behaviour, pair bonding behaviour, parental behaviour, and perhaps other, less obvious, types of social behaviour. Each one of these types of behaviour is most likely stimulated by one particular (nonapeptide) neural circuit. We expect that interdisciplinary integration of ultimate- and proximate-level perspectives will continue to improve our understanding of social behaviour in the future [7]. Additional comparative studies that include a detailed understanding of social organisation, isolate a single aspect of behaviour, such as pair bonding, and employ a fine resolution of brain anatomy have a promising outlook for identifying discrete nonapeptide circuits and characterising their particular contributions to larger, emergent forms of social behaviour.

## Competing interests

We acknowledge financial support for this publication by the German Research Foundation (FOR 1232) and the Open Access Publication Fund of Bielefeld and Muenster University.

The authors declare no competing interests.

## Authors’ contributions

RGO, RMH, and HAH wrote and approved the manuscript.

## References

[B1] Tinbergen N On aims and methods of ethology Z Tierpsychol 1963 20 410 433

[B2] Emlen ST Oring LW Ecology, sexual selection, and the evolution of mating systems Science 1977 197 215 223 10.1126/science.327542 327542

[B3] Goodson JL Nonapeptides and the evolutionary patterning of sociality Prog Brain Res 2008 170 3 15 18655867 10.1016/S0079-6123(08)00401-9 PMC2570786

[B4] Hofmann HA The neuroendocrine action potential Horm Behav 2010 58 555 562 10.1016/j.yhbeh.2010.06.012 20600047

[B5] Ophir AG Towards meeting Tinbergen's challenge Horm Behav 2011 60 22 27 10.1016/j.yhbeh.2011.03.012 21497602

[B6] O’ Connell LA Hofmann HA Genes, hormones, and circuits: an integrative approach to the study the evolution of social behavior Front Neuroendocrinol 2011 32 320 325 10.1016/j.yfrne.2010.12.004 21163292

[B7] Hofmann HA Beery AK Blumstein DT Couzin ID Earley RL Hayes LD An evolutionary framework for studying mechanisms of social behavior Trends Ecol Evol 2014 29 581 589 10.1016/j.tree.2014.07.008 25154769

[B8] Huntingford FA Turner AK Animal conflict 1987 London: Chapman and Hall

[B9] Grand TC Grant JWA Spatial predictability of food influences its monopolization and defence by juvenile convict cichlids Anim. Behav 1994 47 91 100 10.1006/anbe.1994.1010

[B10] Gray SJ Jensen SP Hurst JL Structural complexity of territories: preference, use of space and defence in commensal house mice *Mus domesticus* Anim Behav 2000 60 765 772 10.1006/anbe.2000.1527 11124874

[B11] Warner RR Hoffman SG Population density and the economics of territorial defence in a coral reef fish Ecol 1980 61 772 780 10.2307/1936747

[B12] Grant JWA Godin J-GJ, editor. Territoriality Behavioural ecology of teleost fishes 1997 Oxford: Oxford University Press 81 103

[B13] Brown JL The evolution of diversity in avian territorial systems Wilson Bull 1964 76 160 169

[B14] Grant JWA Whether or not to defend? The influence of resource distribution Mar Behav Physiol 1993 22 137 153 10.1080/10236249309378862

[B15] Maher CR Lott DF A review of ecological determinants of territoriality within vertebrate species Amer Mid Natural 2000 143 1 29 10.1674/0003-0031(2000)143[0001:AROEDO]2.0.CO;2

[B16] Oldfield RG Aggression and welfare in a common aquarium fish, the Midas cichlid J Appl Anim Wel Sci 2011 14 340 360 10.1080/10888705.2011.600664 21932947

[B17] Oldfield RG Behavioral interaction, body size, and sex determination in the Midas cichlid *Amphilophus citrinellus* J Fish Internat 2007 2 242 249

[B18] Fretwell SD Lucas HL Jr On territorial behaviour and other factors influencing habitat distribution in birds. I. Theoretical development Act Biotheoretic 1969 19 16 36 10.1007/BF01601953

[B19] Ens BJ Weissing FJ Drent RH The despotic distribution and deferred maturity: two sides of the same coin Amer Natural 1995 146 625 650 10.1086/285818

[B20] Pitcher TJ Pitcher TJ Functions of shoaling behaviour in teleosts The behaviour of teleost fishes 1986 London: Croom Helm 294 337 10.1007/978-1-4684-8261-4_12

[B21] Hoare DJ Couzin ID Godin J-GJ Krause J Context-dependent group size choice in fish Anim Behav 2004 67 155 164 10.1016/j.anbehav.2003.04.004

[B22] Shuster SM Wade MJ Mating systems and strategies 2003 Princeton: Princeton University Press

[B23] Schradin C Kinahan AA Pillay N Cooperative breeding in groups of synchronously mating females and evolution of large testes to avoid sperm depletion in African striped mice Biol Reprod 2009 81 111 117 10.1095/biolreprod.108.075838 19264699

[B24] Mock DW Fujioka M Monogamy and long-term pair bonding in vertebrates Trends Ecol Evol 1990 5 39 43 10.1016/0169-5347(90)90045-F 21232318

[B25] Barlow GW Uyeno T, Arai R, Taniuchi T, Matsuura K A comparison of monogamy among freshwater and coral reef fishes Indo-Pacific Fish Biology: Proceedings of the Second International Conference on Indo-Pacific Fishes 1986 Tokyo: Ichthyological Society of Japan 767 775

[B26] Whiteman EA Côté IM Monogamy in marine fishes Biol Rev 2004 79 351 375 doi: 10.1017/S1464793103006304 10.1017/S1464793103006304 15191228

[B27] McKaye KR Competition for breeding sites between the cichlid fishes of Lake Jiloa, Nicaragua Ecol 1977 58 291 302 10.2307/1935604

[B28] Hourigan TF Environmental determinants of butterflyfish social systems Envir Biol Fish 1989 25 61 78 10.1007/BF00002201

[B29] Kvarnemo C Ahnesjö I The dynamics of operational sex ratios and competition for mates Trends Ecol Evol 1996 11 404 408 10.1016/0169-5347(96)10056-2 21237898

[B30] Magurran AE The adaptive significance of schooling as an anti-predator defence in fish Ann Zool Fenn 1990 27 51 66

[B31] Zann RA The zebra finch - a synthesis of field and laboratory studies 1996 Oxford: Oxford University Press

[B32] Foster SA Evolution of behavioural phenotypes: influences of ancestry and expression Anim Behav 2013 85 1061 1075 10.1016/j.anbehav.2013.02.008

[B33] Renn SC Schumer ME Genetic accommodation and behavioural evolution: insights from genomic studies Anim Behav 2013 85 1012 1022 10.1016/j.anbehav.2013.02.012

[B34] Krebs CJ Population fluctuations in rodents 2013 Chicago: University of Chicago Press

[B35] Dingemanse NJ Wolf M Between-individual differences in behavioural plasticity within populations: causes and consequences Anim Behav 2013 85 1031 1039 10.1016/j.anbehav.2012.12.032

[B36] Blomberg SP Garland T Tempo and mode in evolution: phylogenetic inertia, adaptation and comparative methods J Evol Biol 2002 15 899 910 10.1046/j.1420-9101.2002.00472.x

[B37] Oldfield RG Mandrekar K Nieves MX Hendrickson DA Chakrabarty P Swanson BO Parental care in the Cuatro Ciénegas cichlid *Herichthys minckleyi* (Teleostei: Cichlidae) Hydrobiol 2015 748 233 257 10.1007/s10750-014-2081-4

[B38] Schradin C Lindholm AK Johannesen J Schoepf I Yuen C-H König B Social flexibility and social evolution in mammals: a case study of the African striped mouse (*Rhabdomys pumilio*) Molec Ecol 2012 21 541 553 10.1111/j.1365-294X.2011.05256.x 21883591

[B39] Young LJ Flanagan-Cato LM Oxytocin, vasopressin and social behavior HormBehav 2012 63 227 462 10.1016/j.yhbeh.2012.02.019 PMC4005251 22443808

[B40] Choleris E Pfaff DW Kavaliers M Oxytocin, vasopressin, and related peptides in the regulation of behavior 2013 Cambridge: Cambridge University Press

[B41] Donaldson ZR Young LJ Oxytocin, vasopressin, and the neurogenetics of sociality Sci 2008 322 900 4 10.1126/science.1158668 18988842

[B42] Ross HE Freeman SM Spiegel LL Ren X Terwilliger EF Young LJ Variation in oxytocin receptor density in the nucleus accumbens has differential effects on affiliative behaviors in monogamous and polygamous voles J Neurosci 2009 29 1312 1318 10.1523/JNEUROSCI.5039-08.2009 19193878 PMC2768419

[B43] Goodson JL Thompson RR Nonapeptide mechanisms of social cognition, behavior and species-specific social systems Curr Opin Neurobiol 2010 20 784 794 10.1016/j.conb.2010.08.020 20850965

[B44] Albers HE The regulation of social recognition, social communication and aggression: vasopressin in the social behavior neural network Horm Behav 2012 61 283 292 10.1016/j.yhbeh.2011.10.007 22079778

[B45] De Vries GJ Buijs RM The origin of vasopressinergic and oxytocinergic innervation of the rat brain with special reference to the lateral septum Brain Res 1983 273 307 317 10.1016/0006-8993(83)90855-7 6311351

[B46] Caffe’ AR Van Leeuwen FW Luiten PGM Vasopressin cells in the medial amygdala of the rat project to the lateral septum and ventral hippocampus J Comp Neurol 1987 261 237 252 10.1002/cne.902610206 3305600

[B47] Freeman SM Young L Choleris E, Pfaff DW, Kavaliers M Oxytocin, vasopressin, and the evolution of mating systems in mammals Oxytocin, vasopressin, and related peptides in the regulation of behaviour 2013 Cambridge: Cambridge University Press 128 147

[B48] Winslow JT Hastings N Carter CS Harbaugh CR Insel TR A role for central vasopressin in pair bonding in monogamous prairie voles Nat 1993 365 545 8 10.1038/365545a0 8413608

[B49] Wang Z Ferris CF De Vries GJ Role of septal vasopressin innervation in paternal behavior in prairie voles (*Microtus ochrogaster*) Proc Nat Acad Sci USA 1994 91 400 404 10.1073/pnas.91.1.400 8278401 PMC42955

[B50] Young LJ Nilsen R Waymire KG MacGregor GR Insel TR Increased affiliative response to vasopressin in mice expressing the V1a receptor from a monogamous vole Nat 1999 400 766 768 10.1038/23475 10466725

[B51] Liu Y Curtis JT Wang Z Vasopressin in the lateral septum regulates pair bond formation in male prairie voles (*Microtus ochrogaster*) Behav Neurosci 2001 115 910 919 11508730 10.1037//0735-7044.115.4.910

[B52] Bester-Meredith JK Young LJ Marler CA Species differences in paternal behavior and aggression in *Peromyscus* and their associations with vasopressin immunoreactivity and receptors Horm Behav 1999 36 25 38 10.1006/hbeh.1999.1522 10433884

[B53] De Vries GJ Sex differences in vasopressin and oxytocin innervation of the brain Prog Brain Res 2008 170 17 27 http://dx.doi.org/10.1016/S0079-6123(08)00402-0 18655868 10.1016/S0079-6123(08)00402-0

[B54] van Anders SM Goldey KL Kuo PX The steroid/peptide theory of social bonds: integrating testosterone and peptide responses for classifying social behavioral contexts Psychoneuroendocrinol 2011 36 1265 1275 10.1016/j.psyneuen.2011.06.001 21724336

[B55] Dore R Phan A Clipperton-Allen AE Kavaliers M Choleris E Choleris E, Pfaff DW, Kavaliers M The involvement of oxytocin and vasopressin in social recognition and social learning: interplay with the sex hormones Oxytocin, vasopressin, and related peptides in the regulation of behaviour 2013 Cambridge: Cambridge University Press 232 255

[B56] Ferguson JN Young LJ Insel TR The neuroendocrine basis of social recognition FrontNeuroendocrinol 2002 23 200 224 11950245 10.1006/frne.2002.0229

[B57] Temeles EJ The role of neighbors in territorial systems: when are they ‘dear enemies’? Anim Behav 1994 47 339 350 10.1006/anbe.1994.1047

[B58] Bielsky IF Hu SB Ren X Terwilliger EF Young LJ The V1a vasopressin receptor is necessary and sufficient for normal social recognition: a gene replacement study Neuron 2005 47 503 513 10.1016/j.neuron.2005.06.031 16102534

[B59] Weitekamp CA Hofmann HA Evolutionary themes in the neurobiology of social cognition Curr Opin Neurobiol 2014 28 22 7 24981873 10.1016/j.conb.2014.06.005

[B60] Maney DL Erwin KL Goode CT Neuroendocrine correlates of behavioral polymorphism in white-throated sparrows Horm Behav 2005 48 196 206 10.1016/j.yhbeh.2005.03.004 15878570

[B61] Goodson JL Wang Y Valence-sensitive neurons exhibit divergent functional profiles in gregarious and asocial species Proc Nat Acad Sci USA 2006 103 17013 7 10.1073/pnas.0606278103 17071744 PMC1636570

[B62] Grober MS George AA Watkins KK Carneiro LA Oliveira RF Forebrain AVT and courtship in a fish with male alternative reproductive tactics Brain Res Bull 2002 57 423 5 10.1016/S0361-9230(01)00704-3 11923002

[B63] Lema SC Slane MA Salvesen KE Godwin J Variation in gene transcript profiles of two V1a-type arginine vasotocin receptors among sexual phases of bluehead wrasse (*Thalassoma bifasciatum*). Gen Comp Endocrinol 2012 179 451 464 10.1016/j.ygcen.2012.10.001 23063433

[B64] Kleszczyńska A Sokołowska E Kulczykowska E Variation in brain arginine vasotocin (AVT) and isotocin (IT) levels with reproductive stage and social status in males of three-spined stickleback (*Gasterosteus aculeatus*) Gen Comp Endocrinol 2012 175 290 296 10.1016/j.ygcen.2011.11.022 22137910

[B65] Insel TR Wang ZX Ferris CF Patterns of brain vasopressin receptor distribution associated with social organization in microtine rodents J Neurosci 1994 14 5381 5392 8083743 10.1523/JNEUROSCI.14-09-05381.1994 PMC6577077

[B66] Bester-Meredith JK Martin PA Marler CA Manipulations of vasopressin alter aggression differently across testing conditions in monogamous and non-monogamous *Peromyscus* mice Aggress Behav 2005 31 189 199 10.1002/ab.20075

[B67] Semsar K Klomberg KF Marler C Arginine vasotocin increases calling-site acquisition by nonresident male grey treefrogs Anim Behav 1998 56 983 7 10.1006/anbe.1998.0863 9790709

[B68] Kelly AM Kingsbury MA Hoffbuhr K Schrock SE Waxman B Kabelik D Vasotocin neurons and septal V1a-like receptors potently modulate songbird flocking and responses tonovelty Horm Behav 2011 60 12 21 10.1016/j.yhbeh.2011.01.012 21295577 PMC3106146

[B69] Veenema AH Beiderbeck DI Lukas M Neumann ID Distinct correlations of vasopressin release within the lateral septum and the bed nucleus of the stria terminalis with the display of intermale aggression Horm Behav 2010 58 273 281 10.1016/j.yhbeh.2010.03.006 20298693

[B70] Pagani JH Wersinger SR Young WS III Choleris E, Pfaff DW, Kavaliers M The roles of vasopressin and oxytocin in aggression Oxytocin, vasopressin, and related peptides in the regulation of behaviour 2013 Cambridge: Cambridge University Press 193 212

[B71] Ferris CF Albers HE Wesolowski SM Goldman BD Leeman SE Vasopressin injected into the hypothalamus triggers a stereotypic behavior in golden hamsters Sci 1984 224 521 3 10.1126/science.6538700 6538700

[B72] Ferris CF Pollock J Albers HE Leeman SE Inhibition of flank-marking behavior in golden hamsters by microinjection of a vasopressin antagonist into the hypothalamus Neurosci Lett 1985 55 239 243 10.1016/0304-3940(85)90027-8 4039806

[B73] Irvin RW Szot P Dorsa DM Potegal M Ferris CF Vasopressin in the septal area of the golden hamster controls scent marking and grooming Phys Behav 1990 48 693 9 10.1016/0031-9384(90)90213-N 2082369

[B74] Bamshad M Albers E Neural circuitry controlling vasopressin-stimulated scent marking in Syrian hamsters (*Mesocricetus auratus*) J Comp Neurol 1996 369 252 263 10.1002/(SICI)1096-9861(19960527)369:2<252::AID-CNE6>3.0.CO;2-2 8726998

[B75] McGuire B Getz L The nature and frequency of social interactions among free-living prairie voles (*Microtus ochrogaster*) Behav Ecol Sociobiol 1998 43 271 9 10.1007/s002650050491

[B76] McGuire B Getz LL Alternative male reproductive strategies in a natural population of prairie voles (*Microtus ochrogaster*) Act Theriolog 2010 55 261 270 10.4098/j.at.0001-7051.077.2009

[B77] Solomon NS Jacquot JJ Characteristics of resident and wandering prairie voles *Microtus ochrogaster* Canad J Zool 2002 80 951 5 10.1139/z02-053

[B78] Ophir AG Phelps SM Sorin AB Wolff JO Social but not genetic monogamy is associated with greater breeding success in prairie voles Anim Behav 2008 75 1143 1154 10.1016/j.anbehav.2007.09.022

[B79] Ophir AG Wolff JO Phelps SM Variation in neural V1aR predicts sexual fidelity and space use among prairie voles in semi-natural settings Proc Nat Acad Sci USA 2008 105 1249 1254 10.1073/pnas.0709116105 18212120 PMC2234124

[B80] O’ Connell LA Hofmann HA The vertebrate mesolimbic reward system and social behavior network: a comparative synthesis J Comp Neurol 2011 519 3599 3639 10.1002/cne.22735 21800319

[B81] Goodson JL Adkins-Regan E Effect of intraseptal vasotocin and vasoactive intestinal polypeptide infusions on courtship song and aggression in the male zebra finch (*Taeniopygia guttata*) J Neuroendocrinol 1999 11 19 25 9918225 10.1046/j.1365-2826.1999.00284.x

[B82] Goodson JL Lindberg L Johnson P Effects of central vasotocin and mesotocin manipulations on social behavior in male and female zebra finches Horm Behav 2004 45 136 143 10.1016/j.yhbeh.2003.08.006 15019801

[B83] Kabelik D Klatt JD Kingsbury MA Goodson JL Endogenous vasotocin exerts context-dependent behavioral effects in a semi-naturalistic colony environment Horm Behav 2009 56 101 7 10.1016/j.yhbeh.2009.03.017 19341739 PMC2723850

[B84] Goodson JL Territorial aggression and dawn song are modulated by septal vasotocin and vasoactive intestinal polypeptide in male field sparrows (*Spizella pusilla*) Horm Behav 1998 34 67 77 10.1006/hbeh.1998.1467 9735230

[B85] Goodson JL Vasotocin and vasoactive intestinal polypeptide modulate aggression in a territorial songbird, the violet-eared waxbill (Estrildidae: *Uraeginthus granatina*) Gen Comp Endocrinol 1998 111 233 244 10.1006/gcen.1998.7112 9679095

[B86] Kelly AM Goodson JL Behavioral relevance of species-specific vasotocin anatomy in gregarious finches Front Neurosci 2013 7 242 doi: 10.3389/fnins.2013.00242 24381536 10.3389/fnins.2013.00242 PMC3865460

[B87] Kime NM Whitney TK Davis ES Marler CA Arginine vasotocin promotescalling behavior and call changes in male túngara frogs Brain Behav Evol 2007 69 254 265 10.1159/000099613 17299257

[B88] Kabelik D Alix VC Burford ER Singh LJ Aggression- and sex-induced neural activity across vasotocin populations in the brown anole Horm Behav 2013 63 437 446 10.1016/j.yhbeh.2012.11.016 23201179

[B89] Kunte K Natural history and reproductive behavior of *Nyctibatrachus cf. humayuni* (Family Ranidae: Anura) Herp Rev 2004 35 137 140

[B90] Rivas JA Burghardt GM Snake mating systems, behavior, and evolution: the revisionary implications of recent findings J Comp Psychol 2005 119 447 454 16366778 10.1037/0735-7036.119.4.447

[B91] Brown JL Morales V Summers K A key ecological trait drove the evolution of biparental care and monogamy in an amphibian Amer Natural 2010 175 436 446 10.1086/650727 20180700

[B92] Eyck GR Ten Arginine vasotocin activates advertisement calling and movement in the territorial Puerto Rican frog *Eleutherodactylus coqui* Horm Behav 2005 47 223 9 10.1016/j.yhbeh.2004.10.005 15664026

[B93] Marler CA Boyd SK Wilczynski W Forebrain arginine vasotocin correlates of alternative mating strategies in cricket frogs Horm Behav 1999 36 53 61 10.1006/hbeh.1999.1524 10433886

[B94] Godwin J Thompson R Nonapeptides and social behavior in fishes Horm Behav 2012 61 230 8 10.1016/j.yhbeh.2011.12.016 22285647

[B95] Dewan AK Maruska KP Tricas TC Arginine vasotocin neuronal phenotypes among congeneric territorial and shoaling reef butterflyfishes: species, sex, and reproductive season comparisons J Neuroendocrinol 2008 20 1382 1394 10.1111/j.1365-2826.2008.01798.x 19094086

[B96] Greenwood AK Wark AR Fernald RD Hofmann HA Expression of arginine vasotocin in distinct preoptic regions is associated with dominant and subordinate behavior in an African cichlid fish Proc Roy Soc Lond B 2008 275 2393 2402 10.1098/rspb.2008.0622 PMC2603226 18628117

[B97] Thompson RR Walton JC Vasotocin immunoreactivity in goldfish brains: characterizing primitive circuits associated with social regulation Brain Behav Evol 2009 73 153 164 10.1159/000219485 19468212

[B98] Ramallo MR Grober M Cánepa MM Morandini L Pandolfi M Arginine-vasotocin expression and participation in reproduction and social behavior in males of the cichlid fish *Cichlasoma dimerus* Gen Comp Neuroendocrinol 2012 179 221 231 10.1016/j.ygcen.2012.08.015 22940647

[B99] Larson ET O’ Malley DM Melloni RH Jr Aggression and vasotocin are associated with dominant-subordinate relationships in zebrafish Behav Brain Res 2006 167 94 102 10.1016/j.bbr.2005.08.020 16213035

[B100] Santangelo N Bass AH Individual behavioral and neuronal phenotypes for arginine vasotocin mediated courtship and aggression in a territorial teleost Brain Behav Evol 2010 75 282 291 10.1159/000316867 20693783

[B101] Dewan AK Tricas TC Arginine vasotocin neuronal phenotypes and their relationship to aggressive behavior in the territorial monogamous multiband butterflyfish *Chaetodon multicinctus* Brain Res 2011 1401 74 84 21676381 10.1016/j.brainres.2011.05.029

[B102] Lema SC Nevitt GA Variation in vasotocin immunoreactivity in the brain of recently isolated populations of a Death Valley pupfish *Cyprinodon nevadensis* Gen Comp Endocrinol 2004 135 300 9 10.1016/j.ygcen.2003.10.006 14723882

[B103] Iwata E Nagai Y Sasaki H Social rank modulates brain arginine vasotocin immunoreactivity in false clown anemonefish (*Amphiprion ocellaris*) Fish Physiol Biochem 2010 36 337 345 10.1007/s10695-008-9298-y 19116767

[B104] Aubin-Horth N Desjardins JK Martei YM Balshine S Hofmann HA Masculinized dominant females in a cooperatively breeding species Molec Ecol 2007 16 1349 1358 10.1111/j.1365-294X.2007.03249.x 17391260

[B105] Renn SCP Aubin-Horth N Hofmann HA Fish and chips: functional genomics of social plasticity in an African cichlid fish J Exper Biol 2008 211 3041 3056 10.1242/jeb.018242 18775941 PMC3728697

[B106] Filby AL Paull GC Hickmore TF Tyler CR Unravelling the neurophysiological basis of aggression in a fish model BMC Genom 2010 11 498 10.1186/1471-2164-11-498 PMC2996994 20846403

[B107] Almeida O Gozdowska M Kulczykowska E Oliveira RF Brain levels of arginine vasotocin and isotocin in dominant and subordinate males of a cichlid fish Horm Behav 2012 61 212 7 10.1016/j.yhbeh.2011.12.008 22206822

[B108] Oldfield RG Hofmann HA Neuropeptide regulation of social behavior in a monogamous cichlid fish Physiol Behav 2011 102 296 303 10.1016/j.physbeh.2010.11.022 21112347

[B109] Santangelo N Bass AH New insights into neuropeptide modulation of aggression: field studies of arginine vasotocin in a territorial tropical damselfish Proc Roy Soc B 2006 273 3085 3092 10.1098/rspb.2006.3683 PMC1679891 17015351

[B110] Backström T Winberg S Arginine-vasotocin influence on aggressive behavior and dominance in rainbow trout Physiol Behav 2009 96 470 5 10.1016/j.physbeh.2008.11.013 19087884

[B111] Bastian J Schniederjan S Nguyenkim J Arginine vasotocin modulates a sexually dimorphic communication behavior in the weakly electric fish *Apteronotus leptorhynchus* J Exper Biol 2001 204 1909 1923 11441033 10.1242/jeb.204.11.1909

[B112] Lema SC Nevitt GA Exogenous vasotocin alters aggression during agonistic exchanges in male Amargosa River pupfish (*Cyprinodon nevadensis amargosae*) Horm Behav 2004 46 628 637 10.1016/j.yhbeh.2004.07.003 15555505

[B113] Godwin J Sawby R Warner RR Crews D Grober MS Hypothalamic arginine vasotocin mRNA abundance variation across sexes and with sex change in a coral reef fish Brain Behav Evol 2000 55 77 84 10.1159/000006643 10838478

[B114] Lema SC Identification of multiple vasotocin receptor cDNAs in teleost fish: sequences, phylogenetic analysis, sites of expression, and regulation in the hypothalamus and gill in response to hyperosmotic challenge Molec Cell Endocrinol 2010 321 215 230 10.1016/j.mce.2010.02.015 20167249

[B115] Semsar K Kandel FL Godwin J Manipulations of the AVT system shift social status and related courtship and aggressive behavior in the bluehead wrasse Horm Behav 2001 40 21 31 10.1006/hbeh.2001.1663 11467881

[B116] Semsar K Godwin J Multiple mechanisms of phenotype development in the bluehead wrasse Horm Behav 2004 45 345 353 10.1016/j.yhbeh.2004.01.003 15109909

[B117] Foran CM Bass AH Preoptic AVT immunoreactive neurons of a teleost fish with alternative reproductive tactics Gen Comp Endocrinol 1998 111 271 282 10.1006/gcen.1998.7113 9707473

[B118] Miranda JA Oliveira RF Carneiro LA Santos RS Grober MS Neurochemical correlates of male polymorphism and alternative reproductive tactics in the Azorean rock-pool blenny *Parablennius parvicornis* Gen Comp Endocrinol 2003 132 183 9 10.1016/S0016-6480(03)00063-7 12812764

[B119] Oliveira RF Ros AF Gonçalves DM Intra-sexual variation in male reproduction in teleost fish: a comparative approach Horm Behav 2005 48 430 9 10.1016/j.yhbeh.2005.06.002 16045912

[B120] Carneiro LA Oliveira RF Canário AVM Grober MS The effect of arginine vasotocin on courtship behaviour in a blenniid fish with alternative reproductive tactics Fish Physiol Biochem 2003 28 241 3 10.1023/B:FISH.0000030542.31395.8a

[B121] Hölldobler B Territoriality in ants Proc Amer Philosoph Soc 1979 123 211 8

[B122] Shellman-Reeve JS Courting strategies and conflicts in a monogamous, biparental termite Proc Roy Soc B 1999 266 137 144 10.1098/rspb.1999.0613

[B123] Scott MP Resource defense and juvenile hormone: the “challenge hypothesis” extended to insects Horm Behav 2006 49 276 281 10.1016/j.yhbeh.2005.07.003 16087184

[B124] Gruber CW Muttenthaler M Discovery of defense- and neuropeptides in social ants by genome-mining PLoS ONE 2012 7 e32559 doi: 10.1371/journal.pone.0032559 10.1371/journal.pone.0032559 22448224 PMC3308954

[B125] Gruber CW Physiology of invertebrate oxytocin and vasopressin neuropeptides Exper Physiol 2014 99 55 61 10.1113/expphysiol.2013.072561 23955310 PMC3883647

[B126] Stafflinger E Hansen KK Hauser F Schneider M Cazzamali G Williamson M Cloning and identification of an oxytocin/vasopressin-like receptor and its ligand from insects Proc Nat Acad Sci USA 2008 105 3262 7 10.1073/pnas.0710897105 18316733 PMC2265169

[B127] Fujino Y Nagahama T Oumi T Ukena K Morishita F Furukawa Y Possible functions of oxytocin/vasopressin-super family peptides in annelids with special reference to reproduction and osmoregulation J Exper Zool 1999 284 401 6 10.1002/(SICI)1097-010X(19990901)284:4<401::AID-JEZ6>3.0.CO;2-U 10451417

[B128] Wagenaar DA Hamilton MS Huang T Kristan WB French KA A hormone-activated central pattern generator for courtship Curr Biol 2010 20 487 495 10.1016/j.cub.2010.02.027 20226670 PMC2858972

[B129] Bardou I Leprince J Chichery R Vaudry H Agin V Vasopressin/oxytocin-related peptides influence long-term memory of a passive avoidance task in the cuttlefish *Sepia officinalis* Neurobiol Learn Mem 2010 93 240 7 10.1016/j.nlm.2009.10.004 19857582

[B130] Garland T Adolph SC Why not to do 2-species comparative studies – limitations on inferring adaptation Physiol Zool 1994 67 797 828

[B131] Getz LL McGuire B Pizzuto T Hofmann JE Frase B Social organization of the prairie vole *Microtus ochrogaster* J Mamm 1993 74 44 58 10.2307/1381904

[B132] Madison DM Space use and social structure in meadow voles Behav Ecol Sociobiol 1980 7 65 71 10.1007/BF00302520

[B133] Wang Z Species differences in the vasopressin-immunoreactive pathways in the bed nucleus of the stria terminalis and medial amygdaloid nucleus in prairie voles (*Microtus ochrogaster*) and meadow voles (*Microtus pennsylvanicus*) Behav Neurosci 1995 109 305 311 7619320 10.1037//0735-7044.109.2.305

[B134] Jannett FJ Jr Sex ratios in high-density populations of the montane vole *Microtus montanus*, and the behavior of territorial males Behav Ecol Sociobiol 1981 8 297 307 10.1007/BF00299530

[B135] Wang ZX Young LJ Liu Y Insel TR Species differences in vasopressin receptor binding are evident early in development: Comparative anatomic studies in prairie and montane voles J Comp Neurol 1997 378 535 546 10.1002/(SICI)1096-9861(19970224)378:4<535::AID-CNE8>3.0.CO;2-3 9034909

[B136] Young LJ Winslow JT Nilsen R Insel TR Species differences in V1a receptor gene expression in monogamous and nonmonogamous voles: behavioral consequences Behav Neurosci 1997 111 599 605 9189274 10.1037//0735-7044.111.3.599

[B137] Ribble DO Salvioni M Social organization and nest co-occupancy in *Peromyscus californicus*, a monogamous rodent Behav Ecol Sociobiol 1990 26 9 16 10.1007/BF00174020

[B138] Svihla A A comparative life history study of the mice of the genus *Peromyscus* Misc Pub Mus Zool Univ Mich 1932 24 1 39

[B139] Metzgar LH Behavioral population regulation in the woodmouse *Peromyscus leucopus* Amer Mid Natural 1971 86 434 448 10.2307/2423635

[B140] Wolff JO The effects of density, food, and interspecific interference on home range size in *Peromyscus leucopus* and *Peromyscus maniculatus* Can J Zool 1985 63 2657 2662 10.1139/z85-397

[B141] Wolff JO Life history strategies of white-footed mice (*Peromyscus leucopus*) Virg J Sci 1986 37 208 220

[B142] Wolff JO Cicirello DM Mobility versus territoriallity: alternative reproductive strategies in white-footed mice Anim Behav 1990 39 1222 4 10.1016/S0003-3472(05)80799-7

[B143] Blair WF A study of prairie deer-mouse populations in Southern Michigan Amer Mid Natural 1940 24 273 305 10.2307/2420931

[B144] Insel TR Gelhard R Shapiro LE The comparative distribution of forebrain receptors for neurohypophyseal peptides in monogamous and polygamous mice Neurosci 1991 43 623 630 10.1016/0306-4522(91)90321-E 1656322

[B145] Wang Z Zhou L Hulihan T Insel TR Immunoreactivity of central vasopressin and oxytocin pathways in microtine rodents: a quantitative comparative study J Comp Neurol 1996 366 726 737 10.1002/(SICI)1096-9861(19960318)366:4<726::AID-CNE11>3.0.CO;2-D 8833119

[B146] Turner LM Young AR Römpler H Schöneberg T Phelps SM Hoekstra HE Monogamy evolves through multiple mechanisms: evidence from V1aR in deer mice Molec Biol Evol 2010 27 1269 1278 10.1093/molbev/msq013 20097658

[B147] Goodson JL Evans AK Wang Y Neuropeptide binding reflects convergent and divergent evolution in species-typical group sizes Horm Behav 2006 50 223 236 10.1016/j.yhbeh.2006.03.005 16643915 PMC2570780

[B148] Ho JM Murray JH Demas GE Goodson JL Vasopressin cell groups exhibit strongly divergent responses to copulation and male–male interactions in mice Horm Behav 2010 58 368 377 10.1016/j.yhbeh.2010.03.021 20382147 PMC4195792

[B149] Dewan AK Ramey ML Tricas TC Arginine vasotocin neuronal phenotypes, telencephalic fiber varicosities, and social behavior in butterflyfishes (Chaetodontidae): potential similarities to birds and mammals Horm Behav 2011 59 56 66 10.1016/j.yhbeh.2010.10.002 20950619

[B150] Oldfield RG Harris R Hendrickson DA Hofmann HA Arginine vasotocin and androgen pathways are associated with mating system variation in North American cichlid fishes Horm Behav 2013 64 44 52 10.1016/j.yhbeh.2013.04.006 23644171

[B151] Dantzer R Koob G Bluthe R Le Moal M Septal vasopressin modulates social memory in male rats Brain Res 1988 457 143 7 10.1016/0006-8993(88)90066-2 3167559

[B152] Veenema AH Neuman ID Central vasopressin and oxytocin release: regulation of complex social behaviours Prog Brain Res 2008 170 261 276 18655888 10.1016/S0079-6123(08)00422-6

[B153] Thompson RR Walton JC Peptide effects on social behavior: effects of vasotocin and isotocin on social approach behavior in male goldfish (*Carassius auratus*) Behav Neurosci 2004 118 620 6 15174940 10.1037/0735-7044.118.3.620

[B154] Thompson RR Walton JC Bhalla R George KC Beth EH A primitive social circuit: vasotocin-substance P interactions modulate social behavior through a peripheral feedback mechanism in goldfish Eur J Neurosci 2008 27 2285 2293 10.1111/j.1460-9568.2008.06210.x 18445219

[B155] Campbell P Ophir AG Phelps SM Central vasopressin and oxytocin receptor distributions in two species of singing mice J Comp Neurol 2009 516 321 333 10.1002/cne.22116 19637308

[B156] Kline RJ O’ Connell LA Hofmann HA Holt GJ Khan IA Immunohistochemical distribution of an AVT V1a receptor in the brain of a sex changing fish *Epinephelus adscensionis* J Chem Neuroanat 2011 42 72 88 10.1016/j.jchemneu.2011.06.005 21723386

[B157] Leung CH Abebe DF Earp SE Goode CT Grozhik AV Mididoddi P Neural distribution of vasotocin receptor mRNA in two species of songbird Endocrinol 2011 152 4865 4881 10.1210/en.2011-1394 PMC6590851 22067316

[B158] Huffman LS O’ Connell LA Kenkel CD Kline RJ Khan IA Hofmann HA Distribution of nonapeptide systems in the forebrain of an African cichlid fish *Astatotilapia burtoni* J Chem Neuroanat 2012 44 86 97 10.1016/j.jchemneu.2012.05.002 22668656

[B159] O’ Connell LA Hofmann HA Evolution of a vertebrate social decision-making network Sci 2012 336 1154 7 10.1126/science.1218889 22654056

[B160] Goodson JL Kabelik D Dynamic limbic networks and social diversity in vertebrates: from neural context to neuromodulatory patterning Front Neuroendocrinol 2009 30 429 441 10.1016/j.yfrne.2009.05.007 19520105 PMC2763925

[B161] Goodson JL Evans AK Lindberg L Chemoarchitectonic subdivisions of the songbird septum and a comparative overview of septum chemical anatomy in jawed vertebrates J Comp Neurol 2004 473 293 314 10.1002/cne.20061 15116393 PMC2576523

[B162] Goodson JL Bass AH Social behavior functions and related anatomical characteristics of vasotocin/vasopressin systems in vertebrates Brain Res Rev 2001 35 246 265 10.1016/S0165-0173(01)00043-1 11423156

[B163] Young LJ Wang Z The neurobiology of pair bonding Nat Neurosci 2004 7 1048 1054 10.1038/nn1327 15452576

[B164] Lim MM Wang Z Olazabal DE Ren X Terwilliger EF Young LJ Enhanced partner preference in a promiscuous species by manipulating the expression of a single gene Nat 2004 429 754 7 10.1038/nature02539 15201909

[B165] Ganz J Kaslin J Freudenreich D Machate A Geffarth M Brand M Subdivisions of the adult zebrafish subpallium by molecular marker analysis J Comp Neurol 2011 520 633 655 21858823 10.1002/cne.22757

[B166] Ophir AG Gessel A Zheng D-J Phelps SM Oxytocin receptor density is associated with male mating tactics and social monogamy Horm Behav 2012 63 445 453 22285648 10.1016/j.yhbeh.2012.01.007 PMC3312950

[B167] O’ Connell LA Matthews BJ Hofmann HA Isotocin regulates paternal care in a monogamous cichlid fish Horm Behav 2012 61 725 733 10.1016/j.yhbeh.2012.03.009 22498693

[B168] Davies NB Sexual conflict and the polygamy threshold Anim Behav 1989 38 226 234 10.1016/S0003-3472(89)80085-5

[B169] Beery AK Lacey EA Francis DD Oxytocin and vasopressin receptor distributions in a solitary and a social species of tuco-tuco (*Ctenomys haigi* and *Ctenomys sociabilis*) J Comp Neurol 2008 507 1847 1859 10.1002/cne.21638 18271022

[B170] Song Z McCann KE McNeill JK Larkin TE Huhman KL Albers HE Oxytocin induces social communication by activating arginine-vasopressin V1a receptors and not oxytocin receptors Psychoneuroendocrinol 2014 50 14 19 25173438 10.1016/j.psyneuen.2014.08.005 PMC4252597

[B171] Clutton-Brock TH Review lecture: mammalian mating systems Proc Roy Soc Lond B 1989 236 339 372 10.1098/rspb.1989.0027 2567517

[B172] Heske EJ Ostfeld RS Sexual dimorphism in size, relative size of testes, and mating systems in North American voles J Mamm 1990 71 510 9 10.2307/1381789

[B173] Kellogg KA Markert JA Stauffer JR Kocher TD Microsatellite variation demonstrates multiple paternity in lekking cichlid fishes from Lake Malawi, Africa Proc Roy Soc Lond B 1995 260 79 84 10.1098/rspb.1995.0062

[B174] McKaye KR Svata M Stauffer JR Jr Bower size and male reproductive success in a cichlid fish lek Amer Natural 1990 135 597 613 10.1086/285064

